# Cell-Type Heterogeneity in Adipose Tissue Is Associated with Complex Traits and Reveals Disease-Relevant Cell-Specific eQTLs

**DOI:** 10.1016/j.ajhg.2019.03.025

**Published:** 2019-05-23

**Authors:** Craig A. Glastonbury, Alexessander Couto Alves, Julia S. El-Sayed Moustafa, Kerrin S. Small

**Affiliations:** 1 Department of Twin Research and Genetic Epidemiology, King’s College London, London SE1 7EH, UK

**Keywords:** eQTL, genetics, genomics, interactions, cell type composition, transcriptomics, GTEx, TwinsUK, adipose, obesity

## Abstract

Adipose tissue is an important endocrine organ with a role in many cardiometabolic diseases. It is comprised of a heterogeneous collection of cell types that can differentially impact disease phenotypes. Cellular heterogeneity can also confound -omic analyses but is rarely taken into account in analysis of solid-tissue transcriptomes. Here, we investigate cell-type heterogeneity in two population-level subcutaneous adipose-tissue RNA-seq datasets (TwinsUK, n = 766 and the Genotype-Tissue Expression project [GTEx], n = 326) by estimating the relative proportions of four distinct cell types (adipocytes, macrophages, CD4+ T cells, and micro-vascular endothelial cells). We find significant cellular heterogeneity within and between the TwinsUK and GTEx adipose datasets. We find that adipose cell-type composition is heritable and confirm the positive association between adipose-resident macrophage proportion and obesity (high BMI), but we find a stronger BMI-independent association with dual-energy X-ray absorptiometry (DXA) derived body-fat distribution traits. We benchmark the impact of adipose-tissue cell composition on a range of standard analyses, including phenotype-gene expression association, co-expression networks, and *cis*-eQTL discovery. Our results indicate that it is critical to account for cell-type composition when combining adipose transcriptome datasets in co-expression analysis and in differential expression analysis with obesity-related traits. We applied gene expression by cell-type proportion interaction models (G × Cell) to identify 26 cell-type-specific expression quantitative trait loci (eQTLs) in 20 genes, including four autoimmune disease genome-wide association study (GWAS) loci. These results identify cell-specific eQTLs and demonstrate the potential of *in silico* deconvolution of bulk tissue to identify cell-type-restricted regulatory variants.

## Introduction

Adipose tissue is the largest endocrine organ in the human body and has a role in the development of insulin resistance, cardiovascular disease, type 2 diabetes, and many other cardiometabolic disorders. Adipose tissue is heterogeneous; it is comprised of an array of cell types including adipocytes, pre-adipocytes, endothelial cells, and several immune cell subtypes.^1^ Adipose tissue cellular composition changes in response to obesity, and it is thought that this change, in particular the marked increase in immune-cell infiltration, contributes to some of the negative health consequences of obesity.^2^, ^3^, ^4^ It is therefore of interest to understand the cellularity of adipose tissue, its variability in the population, and how this affects health and disease.

As a result of the biomedical importance and relatively easy physical accessibility of subcutaneous adipose tissue, a large body of adipose transcriptomic datasets has been generated from multiple studies, including several studies with more than 200 participants.^5^, ^6^, ^7^, ^8^, ^9^, ^10^ To our knowledge, these studies have not assessed the cellular composition of their samples, despite the fact that cellular heterogeneity is a well-established confounder in transcriptomic analysis of bulk tissues.^11^, ^12^, ^13^ Although extensive investigation and methodological development has centered on computationally accounting for cell-type composition in whole blood,^13^ very few studies have investigated the extent of cellular heterogeneity in other tissues and how it impacts -omic-level analyses.^14^ Directly assessing cell-type composition in adipose tissue is challenging; methods such as flow sorting face technical difficulties, including adipocyte rupturing and shared cell-type-specific surface markers, as well as low throughput and high expense when applied to hundreds of samples. Single-cell analysis could overcome some of these considerations; however, the complex logistics of population-level collection of adipose biopsies and the expense of single-cell analysis mean that there is considerable utility in *in silico* deconvolution of -omic profiles generated from bulk adipose tissue.

Here, we utilize *in silico* deconvolution to estimate the relative proportions of four distinct cell types (adipocytes, macrophages, CD4+ T cells, and micro-vascular endothelial cells [MVEC]) in bulk subcutaneous adipose-tissue transcriptomes from two independent datasets: 766 individuals from TwinsUK and 326 post-mortem Genotype-Tissue Expression project (GTEx) donors. We conduct extensive simulations to investigate whether our methods accurately identify the relevant cell types, the range of cell-type detection possible, and robustness to varying levels of noise and unknown cell content (contamination). We find significant cellular heterogeneity within and between these datasets. We recapitulate the well-known cellular hallmark of obesity and find a positive association between adipose-tissue macrophage abundance and body-mass index (BMI), but we identify stronger relationships to dual-energy X-ray absorptiometry (DXA) derived body-fat distribution traits. We assess the impact of adipose cellular heterogeneity on standard -omic analyses, including *cis*-eQTL discovery, co-expression networks, and differential gene-expression studies. Finally, we utilize cell-type composition in interaction models to identify cell-type-specific expression quantitative trait loci (eQTLs) from bulk-tissue transcriptomes that are enriched for genome-wide association study (GWAS) variants and cell-type-relevant enhancers.

## Material and Methods

### RNA-Seq Alignment and Gene Quantification

All (adipose tissue, primary cells, and iPSCs) reference data were aligned, subjected to quality control (QC), and quantified with the same pipeline to ensure comparability. Reads were aligned to the hg19 reference genome with STAR version 2.4.0.1.^15^ All aligned binary sequence alignment maps were then filtered to isolate those containing reads with a mapping quality greater than 10, and among those, only reads that were properly paired and had two or fewer mismatches were kept. Samples were excluded if they had fewer than 10 million reads mapping to known genes or if the sequence data did not correspond to actual genotype data as assessed with the “mbv” mode of QTLtools.^16^ GENCODE annotation v19 gene counts were calculated via featureCounts^17^ and only genes that coded for proteins and did not have retained intron transcripts. All gene counts were transformed into trimmed mean of M-values (TMM), a unit shown to be well suited for an across-cell-type study design and that also accounts for library size differences.^18^, ^19^ Although all protein-coding genes were used for cell-type estimation (20,345 genes) because filtering lowly expressed genes could bias cell-type estimates to highly abundant cells in a given tissue biopsy, genes with at least 0.5 TMM expressed in 90% of samples within a dataset were retained for transcription-wide association and eQTL analysis.

### TwinsUK Dataset

Sub-umbilical, subcutaneous adipose-tissue punch biopsies were collected from female twins from the TwinsUK cohort, as described previously.^5^, ^6^ RNA-seq data are available in EGA (EGA: S00001000805). QC) of the TwinsUK genotypes has been described previously.^6^, ^20^ After QC, 766 TwinsUK samples were available for analysis, of which 720 had available genotypes. The TwinsUK adipose samples had a median age of 60 [38–84] and median BMI of 25 [16–47]. Cell-type proportions of TwinsUK samples are listed in Table S1.

### GTEx RNA-Seq Dataset

RNA-seq FASTQ data for all GTEx v. 6p subcutaneous adipose-tissue samples were downloaded from the database of genotypes and phenotypes (dbGaP). GTEx subcutaneous adipose tissue samples were obtained from the lower legs of post-mortem donors. To ensure comparability, GTEx data were re-aligned and quantified with the same pipeline used by TwinsUK. In addition, gene-expression principal-component analysis (PCA) outliers were removed; outliers were defined by use of k-means clustering (k = 2) fit to the first two expression principal components (PCs). 326 QC’d samples were retained for analysis and are listed with their cell-type proportions in Table S2.

### Reference Cell-Type Data

To create the adipose signature matrix, we utilized reference RNA-seq datasets for each cell type selected from publicly available RNA-seq data from either primary (CD4+ T cells, HMVEC), PSC-derived (adipocytes), or iPSC-derived (macrophages) sources. Reference RNA-seq data were obtained from the Sequence Read Archive (SRA) as raw FASTA files. All datasets are listed in Table S3. One independent set of experiments was used for construction of the adipose tissue signature matrix, and another independent set was used for construction of *in silico* simulated mixtures for testing deconvolution accuracy. To ensure comparability, we aligned reference cell-type data and quantified them by using the same pipeline used for bulk tissue. We were prevented from estimating additional cell types by the unavailability of reference datasets for those cell types at the time of study, a lack of replicates that would ensure stable construction of the signature matrix, or very low frequency in the tissue (e.g., mast cells). Biological replicates of each of the four reference cell types were included.

### Construction of CIBERSORT Adipose Signature Matrix

RNA-seq data obtained from cell types and their biological replicates were constructed into a reference cell-type matrix with *n* rows (genes) and *m* columns (cell types). We also constructed a class file to describe the pairwise comparisons that one must perform between cell types in order to produce the signature matrix.^21^ The signature matrix contains all genes differentially expressed between the cell types at a specified false discovery rate (FDR) (q = 0.30, default). The CIBERSORT analytical tool has the additional benefit that each tissue or mixture is deconvolved with potentially different signature genes. This is due to the algorithm’s implementation of a ν–support vector regression (ν-SVR) step in which only the maximally separating support vectors are retained for the linear regression. ν-SVR also aids in minimizing co-linearity as measured through the matrix condition number (κ), an ideal step during estimation of cell types that are biologically closely related.

### Estimating Cell Types from Bulk Adipose-Tissue RNA-Seq Data

CIBERSORT was used for estimation of cell-type proportions from adipose-tissue RNA-seq samples from both TwinsUK and GTEx.^21^ For signature matrix construction in CIBERSORT, we used the default value of q = 0.30 for the FDR because CIBERSORT’s support vector regression procedure ensures that a subset of genes that maximally separate cell types is present in each individual adipose tissue sample, so it is therefore better to have a lower-false negative rate when detecting the initial set of signature genes. CIBERSORT also reports the condition number (κ) of the signature matrix, a measure of co-linearity and matrix stability. The signature matrix has a low kappa (κ = 3.22), suggesting that a well-conditioned matrix was achieved. CIBERSORT provides a deconvolution p value per sample, calculated from 1,000 bootstrapped permutations.^21^ We required a deconvolution p value < 0.01 corresponding to an FDR of 1%.

### *In Silico* Mixture Simulations

Reference cell types were combined in random proportions to generate 1,000 *in silico* simulated cell mixtures, termed “the ground truth” (S). We generated a mixture matrix (M) by drawing variables (equal to the total number of cells to form a mixture with) from a random uniform distribution normalized to sum to one and multiplied by the reference cell matrix (C):

$$\text{S}\;=\;{\text{CM}}^{\text{T}}$$

S = truth (known simulated proportions)

C = matrix of reference cell expression profiles

M = mixture matrix specifying amount of each cell type [0–1]

A natural amount of noise is introduced into this problem because the reference cell types are obtained from different laboratories that use different sequencing chemistries. This is ideal because the same problem is present for the deconvolution of the real adipose-tissue mixtures, making the simulated data more realistic. However, to make the problem more challenging and to assess the signature matrix’s limit and ability to deal with noise in mixture profiles, we added 10% to 100% scaled randomly distributed Gaussian noise to each simulated sample:

$${\text{y}}_{1}\;={\text{y}}_{\text{0}}\;+\;\text{X}\;+{\text{y}}_{\text{0}}\;\text{S}$$

x = random normal variable with X ∼N (0, 1)

y_0_ = simulated *in silico* mixture

y_1_ = simulated *in silico* mixture with added noise

S = scale factor [0–1]

### GTEx Histology Images and Pathologist Notes

Histological images of GTEx biopsies along with accompanying pathologist notes were obtained from the GTEx web portal. Although the GTEx histology slides were prepared from a piece of material adjacent to the piece utilized for RNA-seq, they are reflective of the overall tissue sample taken.

### Association between Cell-Type Composition and GTEx Covariates

To assess the relationship between GTEx adipose tissue cell proportion estimates and ischemic time, we fit a linear model controlling for age, sex, BMI, and batch against each cell type estimated. Additionally, we performed PCA on the cell-type proportion matrix and assessed whether ischemic time was correlated to any one of the first three PCs. Finally, given that GTEx is composed of both male- and female-derived samples, we tested for any presence of sexual dimorphism for each cell type while controlling for BMI, age, ischemic time, and batch.

### Heritability Estimation

Heritability calculations were performed with OpenMx.^22^ We fit a standard additive genetic variance, common environmental factors, and nonshared environment model in which additive genetic, common, and unique environment-variance components were estimated for macrophage and adipocyte proportion between twin pairs.

### Association between Cell-Type Composition and Whole-Body Phenotypes

Association between cell-type proportion and whole-body phenotypes (BMI, body-fat distribution, and age) were conducted in the TwinsUK datasets under linear models (lm) in R. All phenotypes were collected at the time of biopsy. Body-fat distribution measurements of android, gynoid, and visceral fat volume were quantified (n = 652) via dual-energy X-ray absorptiometry (DXA; Hologic QDR 4500 plus) according to the standard manufacturer’s protocol.

### Association between BMI and Gene Expression

Each gene-expression measurement (TMM) was tested as a dependent variable in a linear mixed-effects model that accounted for family structure as previously described in detail.^20^ Independent variables in addition to BMI and macrophage proportion included technical covariates that are well known to have strong effects on RNA-seq gene expression studies (fixed effects: insert-size mode, mean GC content, primer index) (random effects: date of sequencing). Using a single-degree-of-freedom ANOVA, we compared the model fit, which was adjusted for macrophage proportion with the null model but was not adjusted for macrophages.

### Weighted Gene Co-Expression Network Analysis

Signed weighted gene co-expression network analysis (WGCNA) was carried out with WCGNA version 1.62 in R as previously described.^23^ Gene networks have been shown to follow a scale-free topology. WGCNA uses soft thresholding to find modules of highly correlated co-expressed genes. The overall process has been described previously.

### *cis*-eQTL Analysis

For global *cis*-eQTL analysis, each *cis*-window was defined as a 1 MB region around the transcription start site (TSS) of each gene. SNPs with an MAF ≥ 5% were analyzed. We used eigenMT to determine significant associations.^24^ eigenMT calculates the number of effective tests per *cis*-window by performing eigenvalue decomposition and taking the effective number of tests as equal to the eigenvalues that explain 99% of the variance. This procedure has been shown empirically to control the FDR similarly to permutations. All analysis was performed with inverse-rank-normalized gene-expression residuals corrected for experimental covariates.^20^ All analysis was conducted with the MatrixeQTL package.^25^ We obtained probabilistic estimation of expression residuals (PEER)-corrected residuals by correcting for 30 PEER factors.^26^

### Gene-by-Cell Proportion Interaction Modeling

Interaction models were fitted with the “modellinear cross” function in MatrixQTL.^25^ To maximize the power to detect *cis*-eQTLs that are dependent on cell-type proportion, we inferred 30 PEER factors by using inverse-rank normalized gene expression residuals corrected for sequencing date, zygosity, and family structure. Interaction models for relative macrophage proportion were adjusted for the following covariates: 30 PEER factors, mean GC content, insert size, BMI, and age. We also inverse normalized macrophage proportion to ensure normally distributed errors.

## Results

### Accurate Cell-Type Estimates That Are Robust against Unknown Content and Noise

We estimated cell-type proportion in bulk adipose tissue RNA-seq profiles with CIBERSORT, a ν-support vector regression (ν-SVR) method that estimates cell proportions by using gene expression obtained from solid tissues.^21^ CIBERSORT identifies cell-type-specific marker genes from reference cell transcriptome profiles to construct a tissue-specific signature matrix, a set of differentially expressed genes across all cell types, and utilizes this signature matrix to perform the deconvolution step.

To construct the CIBERSORT adipose-tissue signature matrix, we obtained previously published RNA-seq datasets from reference *in vitro* cells, including both primary cells and iPSC-derived cells that are known to be present in subcutaneous adipose tissue; these include adipocytes, macrophages, CD4+ T cells, and microvascular endothelial cells (MVECs) (Table S3). Adipose tissue is comprised of many more cell types than the four we focus on here. Hierarchical clustering of the reference transcriptional profiles recapitulated developmental cellular hierarchy (Figure S1). The final CIBERSORT adipose signature matrix is comprised of 658 genes, including several encoding well-known cell-type-specific markers. Examples include *SCD* (MIM: 604031), *COL1A1* (MIM: 120150), and *ADIPOQ* (MIM: 612556) in adipocytes; *SERPINE1* (MIM: 173360), *MMP1* (MIM: 120353), and *VWF* (MIM: 613160) in endothelial cells; *SPP1* (MIM: 166490), *F13A1* (MIM: 134570), and *CTSC* (MIM: 602365) in macrophages; and *FOS* (MIM: 164810), *TCF7* (MIM: 189908), and *CD3* (MIM: 186780) in T cells. The full signature matrix is included in Table S4.

To test deconvolution ability, accuracy, and robustness to noise, we performed several simulations that are typically used for benchmarking deconvolution accuracy.^19^, ^21^ First, we tested whether the adipose tissue signature matrix can accurately identify the four cell types when applied to a set of independent reference cell-type RNA-seq datasets. All benchmark cell types were estimated with high accuracy: three out of four cell types attained ≥ 99% accuracy in prediction (Table S5). Macrophages (93% accuracy) are particularly difficult to purify, so it is possible that the 6% CD4+ T cells we estimate in the macrophage benchmark sample were present in the original reference macrophage sample.

**Figure 1:**
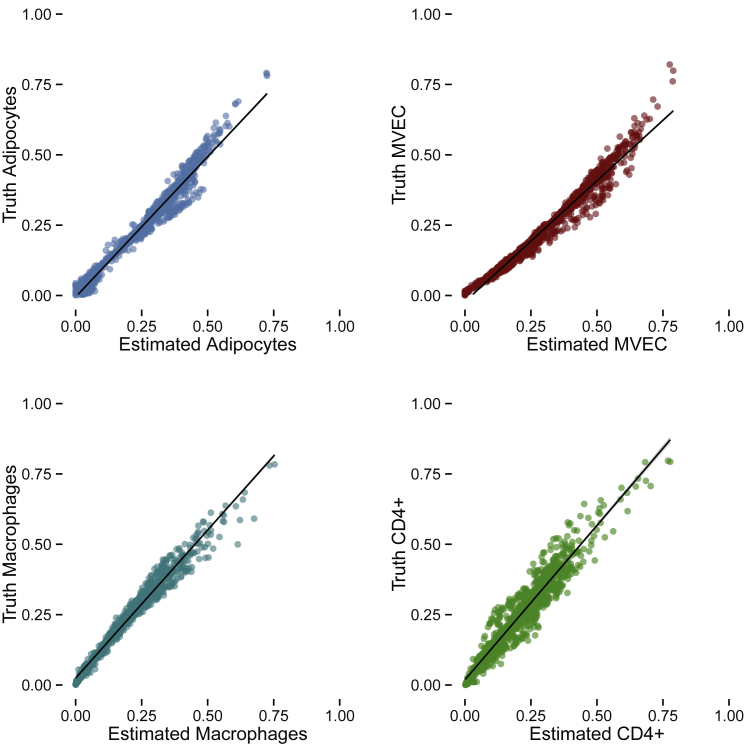
Cell-Type Proportion Is Accurately Estimated in *In Silico* Mixture Simulations Each panel displays cell-type estimation in 1,000 *in silico* mixtures. Each point represents one simulation.

We next assessed the ability to estimate the constituent cell proportions of a mixture of known cell types. We created 1,000 *in silico* mixtures of known proportions of each of the four cell types with the independent reference cell-type datasets (Table S3). Application of CIBERSORT to the *in silico* mixtures yielded highly accurate estimates of cell-type proportion; values for the mean absolute deviation (mAD) of estimated proportions to ground truth ranged from 0.019 to 0.068 (Figure 1). Biopsies can contain contaminant cells from other tissues, which could inflate cell-type proportion estimates if contaminant cells share marker genes with any of the four cell types we are estimating. To test this, we added proportions of smooth-muscle cells, dendritic cells, and neutrophils to the *in silico* mixtures of the four cell types. These cell types can be present in adipose tissue and therefore reflect realistic “contaminant cells.” Neutrophils make up 60%–70% of whole blood and are a likely contaminant. Cell-type prediction was accurate when up to 10% of a sample was composed of contaminant cell types (Figure S2). We note that adipose-resident cells not included in our matrix but with similar expression profiles to that of a reference cell could inflate estimates of that cell type; in particular, the estimated adipocyte proportion might be inflated because of the lack of appropriate reference RNA-seq for adipocyte mesenchymal stem cells and pre-adipocytes. It is likely that the content of unknown cells in the samples is ≤10% given previously published cell-type estimates from adipose tissue,;^27^, ^28^ thus, the adipose tissue signature matrix is robust in estimating cell types from mixtures with some unknown content.

Technical factors during library preparation and sequencing introduce noise in RNA-seq experiments. Therefore, as in previous work, we tested how much noise we could introduce into the simulations and still accurately predict cell-type proportions.^21^ We added 10%, 50%, and 90% Gaussian noise, in addition to the naturally occurring noise present in each of the separate experimentally derived reference RNA-seq datasets. The estimates are robust when up to 10% of the mixture is distorted with noise, and a linear relationship between ground truth and predicted estimates still holds when large amounts of noise are introduced (Figure S3).

### Estimation of Relative Cell-Type Proportions in Bulk Adipose RNA-Seq Datasets

**Figure 2:**
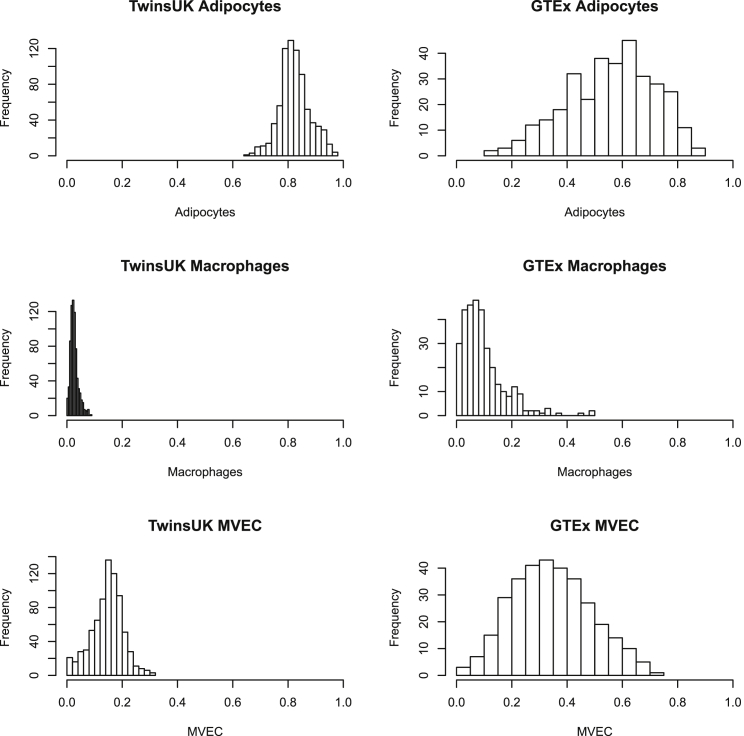
Distribution of Relative Cell-Type Estimates in TwinsUK and GTEx Adipose Samples TwinsUK samples are shown on the left, and GTEx samples are on the right.

We applied CIBERSORT and the adipose-tissue signature matrix to a previously published dataset of 766 subcutaneous adipose-tissue biopsies obtained from female twin participants in TwinsUK.^5^, ^6^ All 766 TwinsUK RNA-seq samples were successfully deconvolved at an FDR of 1%. Adipocytes were the most dominant relative cell type (0.73–0.99), but also showed significant inter-subject variability (Figure 2). Proportions of the other estimated cell types ranged from 0.004–0.22 for macrophages (M1 and M2 combined), 0–0.19 for MVEC, and 0– 0.11 for CD4+ T cells (Figure 2). These estimates agree with previously published studies using flow cytometry (Table S6). Because the vast majority of TwinsUK adipose samples had CD4+ T cell estimates below 1%, we chose to focus on adipocyte, macrophage, and endothelial-cell estimates for downstream analysis. However, we investigated whether there were any distinct differences between individuals who had non-zero CD4+ T cell counts. In total, two individuals had CD4+ proportions >5%, and 24 individuals had CD4+ proportions >1%. Neither BMI nor age differed significantly between these subjects: BMI (BMI_low CD4+_ 26.6, BMI_hi CD4+_ 26.7) and age (Age_low CD4+_ 59.4, Age_hi CD4+_: 60.9).

We next applied CIBERSORT to an independent sample of 326 post-mortem subcutaneous adipose tissue biopsies from the GTEx. In contrast to the samples from TwinsUK, ∼23% of GTEx samples (75/326) failed successful deconvolution (1% FDR), suggesting substantial differences in cell types present in the tissue from the signature matrix. As compared to the TwinsUK samples, the 251 GTEx samples that passed deconvolution had markedly different cell-type composition profiles, including a lower adipocyte fraction (GTEx_median_ = 0.62, TwinsUK_median_ = 0.82), twice as much vasculature (GTEx median MVEC proportion = 0.30, TwinsUK = 0.15), and four times as many macrophages (GTEx median macrophage = 0.08, TwinsUK = 0.02) (Figure 2). Overall, all cell types between GTEx and TwinsUK differed significantly (adipocytes—t statistic = 39.78, p value = 7.77 × 10^−211^; macrophages—t statistic = −22.585, p value = 4.89 × 10^−92^; MVEC—t statistic = −32.02, p value = 5.75 × 10^−157^).

**Figure 3:**
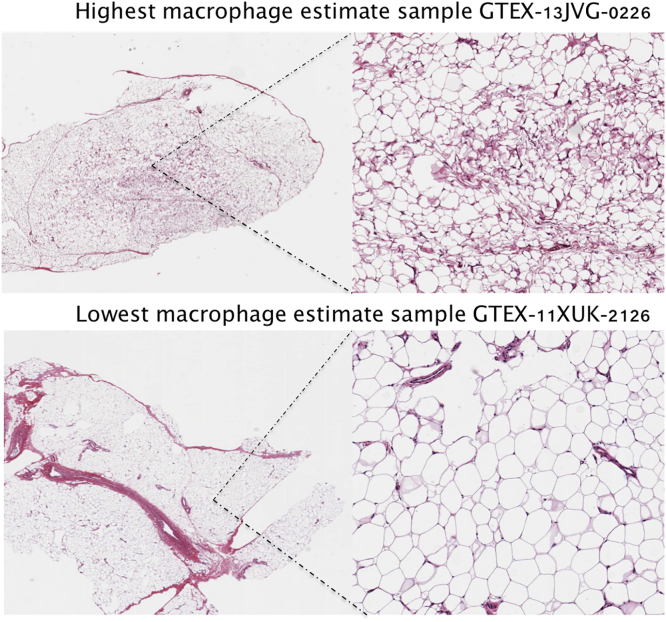
Estimated Cell-Type Composition of GTEx Samples Corresponds to Image Data Histology images from the GTEx adipose samples with the highest (49%) (top) and lowest (0%) (bottom) macrophage estimates are shown. Both whole-biopsy (left) and zoomed-in images (right) are presented. Estimated cell-type composition of all GTEx samples is provided in Table S2.

To assess the GTEx estimates, we investigated whether there were visible histological differences between samples with differential macrophage proportion estimates in GTEx adipose histology slides. We observed concordance between our estimates and visual inspection of the histology slides. We demonstrate this in Figure 3, where the sample with the lowest macrophage proportion (estimated at 0%) is composed primarily of adipocytes with few additional cells present. In stark contrast, the sample with the highest macrophage proportion (estimated at 49%) has substantial vasculature and blood cells present.

To validate the difference in adipocyte proportion between the datasets, we focused on the expression of *ADIPOQ*, which encodes the hormone adiponectin. *ADIPOQ* is expressed highly in adipocytes and pre-adipocytes.^29^ *ADIPOQ* was expressed 4-fold higher in the TwinsUK dataset (median TMM = 3998, expression rank = 19) compared to the GTEx dataset (median TMM = 963, expression rank = 59). The distribution and range of *ADIPOQ* expression varied between the datasets; it followed a normal distribution in the TwinsUK dataset (untransformed, TMM data) and was heavily skewed to the right in the GTEx dataset (Figure S4). *ADIPOQ* expression is very low in some GTEx samples as compared to TwinsUK, which suggests fewer viable adipocytes (GTEx *ADIPOQ*_*min*_ = 3.13 TMM; TwinsUK *ADIPOQ*_*min*_ = 986 TMM) (Figure S4). The *ADIPOQ* results support the CIBERSORT estimates of lower adipocyte proportion in the GTEx samples.

There are several possible additional explanations of why cell-type proportions differ between the TwinsUK and GTEx datasets. The function and metabolic activity of adipose tissue is known to vary between fat depots—markedly between android (above the hip) and gynoid (below the hip) depots. The GTEx adipose samples were obtained via surgical incision from the lower left leg (gynoid fat depot), whereas the TwinsUK samples were derived from punch biopsies from the abdomen (android fat depot). Additional fibrosis is likely to alter the number of viable cells available for sequencing in post-mortem samples; GTEx pathologist notes frequently recorded the presence of large fibrotic regions (up to 60% of a given histology slide). In the GTEX data, ischemic time was not associated with any individual cell type but was correlated with the overall cellular heterogeneity of a sample (p value = 0.0085, r = 0.15), indicating that differences in cell estimates among GTEx samples are partially due to variability in ischemic time between samples. We also note that sex was associated with both adipocytes (p value = 0.028) and MVEC (p value = 0.019) proportion in the GTEx dataset, whereas the TwinsUK population was all female. Given the large disparities in estimated cell composition between the datasets, we chose to focus on the TwinsUK dataset for the following analysis.

### Adipose Cell-Type Proportions Are Heritable

Several studies have demonstrated that the cell-type composition of whole blood is heritable, but the influence of genetics on adipose cell-type composition has not been explored.^30^, ^31^ Using structural equation models, we estimate the narrow-sense heritability (*h*^*2*^) of adipocyte, macrophage, and endothelial cell proportion to be 17%, 30%, and 21% respectively in the TwinsUK data. The heritability of adipose tissue cell composition might be mediated by genetic drivers of whole-body traits, such as BMI, that in turn drive changes in cell-type proportion or might be mediated by local effects within adipose tissue—effects such as rates of adipogenesis or angiogenesis.

### Adipose Tissue Cell-Type Proportion Is Associated with Whole-Body Obesity Traits but Not Age

Macrophage infiltration and abundance in adipose tissue is known to increase with obesity and its associated chronic inflammation.^32^ Recapitulating this finding, we demonstrate a significant correlation between BMI and estimated adipose macrophage proportion in the TwinsUK data (Table 1). To explore the relationship between cell-type composition and body-fat distribution, we used highly accurate dual X-ray absorptiometry (DXA) measures of visceral fat volume (VFAT) and android/gynoid (A/G) ratio in a subset of twins (n = 652) with concurrently measured DXA scans. Despite the smaller sample size, the correlation coefficients between the A/G ratio and visceral fat with relative macrophage estimates were significantly larger than the correlation of BMI with relative macrophage estimates (Table 1). Including BMI as a covariate did not change the associations to DXA-derived traits, indicating that body-fat distribution is associated with adipose tissue cell composition independent of overall adiposity. This finding confirms the importance of macrophage biology in obesity but also suggests that inflammation plays a more prominent role in body-fat distribution than is currently appreciated.

**Table 1:** TwinsUK Macrophage Proportion in Adipose Tissue Is Associated to Obesity-Related Traits but Not Age Suggested edited table:

**Trait**	**r^2^**	**p Value**
**Macrophage**		

BMI	0.22	2.2 × 10^−8^
visceral fat	0.29	4.9 × 10^−15^
visceral fat (BMI adjusted)	0.28	1.9 × 10^−9^
android/gynoid ratio	0.36	1.2 × 10^−16^
android/gynoid ratio (BMI adjusted)	0.35	1.8 × 10^−12^
age	−0.02	n.s

**Adipocyte**		

BMI	0.15	5.7 × 10^−5^
visceral fat	0.13	3.4 × 10^−4^
visceral fat (BMI adjusted)	0.07	0.05
android/gynoid ratio	0.16	2.7 × 10^−5^
android/gynoid ratio (BMI adjusted)	0.11	0.002
age	−0.004	n.s

**MVEC**		

BMI	−0.21	3.62 × 10^−9^
visceral fat	−0.23	6.2 × 10^−10^
visceral fat (BMI adjusted)	−0.14	1 × 10^−4^
android/gynoid ratio	−0.26	6.5 × 10^−13^
android/gynoid ratio (BMI adjusted)	−0.20	1.1 × 10^−7^
age	−0.006	n.s

BMI: body mass index

In contrast to the well-documented association between whole-blood cell-type composition and age, there was no association between age and either macrophage or adipocyte proportion (r = −0.02).^13^ This indicates that adipose cell-type composition is not a major confounder in identification of age-related transcripts,^33^ nor is it differentially methylated in adipose tissue.^34^

### Adjusting for Macrophage Heterogeneity Accounts for 11% of Genes Displaying BMI-Related Differential Expression

**Figure 4:**
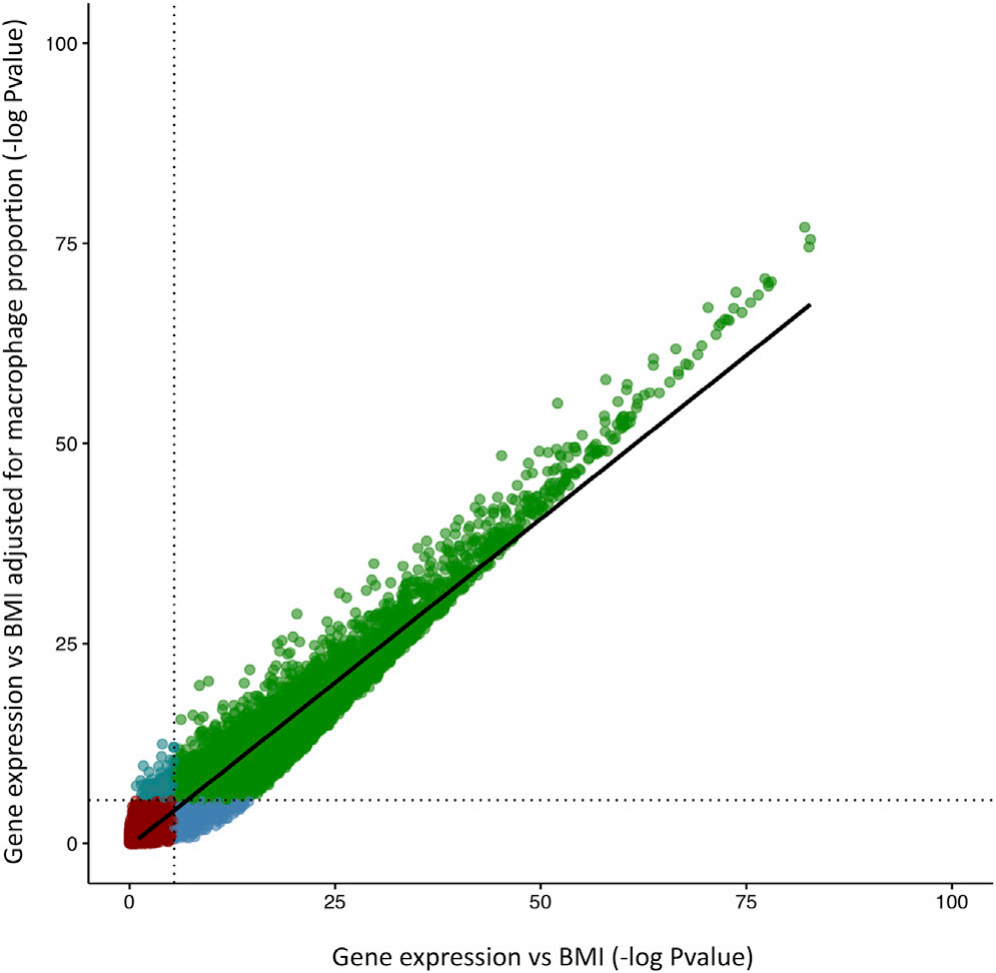
Adjusting for Macrophage Proportion Accounts for 11% of Associations between Gene Expression and BMI Each point represents one gene and is colored as follows: red – significant in neither association; light blue – significant in macrophage-adjusted association only; dark blue – significant in unadjusted association only; and green – significant in both associations.

BMI has a profound effect on adipose tissue gene expression; the majority of the adipose transcriptome is associated with BMI in studies conducted with both microarrays and RNA-seq in independent populations.^7^, ^9^, ^20^ It is unclear how many BMI-associated changes in gene expression are mediated by the changes in cell-type composition that accompany increasing BMI. To address this, we identified associations between gene expression and BMI under two models, one model adjusting and one not adjusting for macrophage proportion. In the first model, we recapitulate previous results with expression of 6,366/14,897 protein-coding genes significantly associated with BMI (Bonferroni-corrected p = 3.56 × 10^−6^). When we adjusted for macrophage proportion, 11% of associations were no longer significant (Figure 4). This demonstrates that although inflammation is an important aspect of obesity etiology, the majority (89%) of BMI-expression associations are likely to be independent of macrophage proportion. An example of one of the 707 genes that are no longer significant after adjustment for macrophage proportion is *CD209* [MIM: 604672] *p*_original_ = 7.72 × 10^−8^, *p*_adj_ = 0.0019), a gene that encodes for a C-type lectin that is found primarily on the surfaces of macrophages and dendritic cells. Additional example genes that were no longer significant include *LILRA2* [MIM: 604812], *MNDA* [MIM: 159553], and *CMKLR1* [MIM: 602351] which are known to be primarily expressed in macrophage and immune cell lineages.^35^, ^36^

### Cell-Type Proportion Explains Major Components of Gene Expression Variance and Co-Variance

Principle component analysis (PCA) is commonly used to understand the sources of gene expression variance. We identified principle components in the TwinsUK samples and correlated them to cell-type proportion estimates. PC1 was correlated with adipocyte and endothelial cell proportion (R = 0.40, p value ≤ 2.2 × 10^−16^; R = 0.41, p value = 2.2 × 10^−16^, respectively). PC2 was negatively correlated with macrophage proportion (R = −0.63, p value ≤ 2.2 × 10^−16^) and positively correlated with endothelial cell proportion (R = 0.21, p value ≤ 3.7 × 10^−9^). PC1 and PC2 cumulatively explained 25% of adipose tissue gene expression variance (Figure S5). This indicates that cell-type heterogeneity at the population level is a major driver of gene expression variation in adipose tissue, and accounting for principle components in downstream analysis should account for some of this variability.

Weighted gene co-expression network analysis (WGCNA) is a widely used technique that uses the correlation structure of global gene expression profiles to construct modules of genes, some of which have been ascribed distinct functional roles or correspond to gene networks. 11 out of 13 WCGNA modules in the TwinsUK data correlated with cell-type proportion (Figure S6, *p.* < 0.0038). The most significant macrophage-proportion-associated module (Pearson’s R = 0.67, p value ≤ 2.2 × 10^−16^) (Figure S6A) recapitulated the macrophage-enriched metabolic network (MEMN), an adipose gene expression signature associated with increasing BMI.^7^, ^37^ The MEMN-green module’s constituent genes were enriched for glycoproteins (p value = 7.1 × 10^−63^), immunity (p value = 1.1 × 10^−23^), and the innate immune response (p value = 4.5 × 10^−12^). Endothelial-cell proportion was positively correlated with the turquoise module (r = 0.41), which was significantly enriched for GO terms related to angiogenesis (p value = 6.4 × 10^−12^). These findings demonstrate that cell-type composition is a major driver of co-expression in bulk-tissue RNA-seq samples and could confound analysis if samples are not matched for cell-type proportion.

### Correction for Macrophage Heterogeneity in Adipose Tissue Increases *Cis*-eQTL Discovery Yield

To determine whether adipose cell-type heterogeneity can confound *cis*-eQTL analysis, we investigated the effect of correcting for cell type in *cis*-eQTL analysis. We implemented a naïve *cis*-eQTL discovery model (we did not adjust for any cell-type proportion) and a separate, macrophage-corrected eQTL model. Adjusting for macrophage heterogeneity among samples led to a modest increase in *cis*-eQTL yield (2.3%) (naive model = 5,531, macrophage-adjusted model = 5,665 SNP-gene pairs, FDR5%). However, it has become standard practice in *cis*-eQTL studies to use gene expression principle components, PEER factors, or other factor-analysis-based methods to estimate and adjust out confounding factors from gene-expression data. To test whether latent factors account for cell-type proportion variability, we re-ran the naive and cell-type-adjusted *cis*-eQTL scans and included adjustment for 30 PEER factors. PEER-factor adjustment achieved a similar increase in *cis*-eQTL yield in the naive and cell-type-adjusted models and resulted in near identical results (naïve PEER = 7,665, macrophage-adjusted PEER = 7,664). This confirms that latent factors capture the cell-type composition differences among adipose samples, as well as many other unmeasured latent factors, but if covariates are known, it is better to adjust with a fully specified model than to estimate latent factors given the known risk of collider bias.^38^

### Identification of Cell-Type-Specific eQTLs from Bulk Tissue

Previous studies have identified cell-type-specific eQTLs in bulk whole-blood expression profiles by fitting gene expression to cell-type interaction models.^39^ We utilized this strategy to detect cell-type-specific *cis*-eQTLs in the TwinsUK adipose data. At a strict Bonferroni-corrected threshold (p value threshold = 1.01 × 10^−9^, based on 49,219,795 association tests in the 1-MB TSS-centered window around 14,897 genes), we identified 26 G × cell-type interactions at 20 unique genes (Table 2) (Figure S7). Twelve gene-SNP pairs had an interaction with macrophage proportion, 10 with endothelial proportion, and four with adipocyte proportion (Table 2). Examples include *MARCO* [MIM: 604870], encoding a macrophage receptor that has collagenous structure and whose expression depends on macrophage proportion, and rs1884841. *TC2N,* which is responsible for the secretion of *VWF* from endothelial cells, and *DEFB1* [MIM: 602056] both showed a positive interaction with adipocytes and a negative interaction with endothelial cells.

**Table 2:** G × Cell-Proportion Interactions Identify Cell-Type-Specific eQTLs from Bulk Adipose-Tissue Gene-Expression Profiles

**Cell Type**	**SNP**	**Gene**	**β**	**p Value**	**Fairfax et al. Stimulus & Proxy SNP**	**GTEx Top eQTL Tissues**	**Regulatory Regions**
macrophage	rs61913538	*CLEC12A*	0.37	8.4 × 10^−25^	naive, rs7313235	WB, adipose, muscle	blood promoter
macrophage	rs1063355	*HLA-DQA1*	0.29	3.0 × 10^−18^	NA	WB, skin, muscle	blood promoter. breast/skin DNAase
macrophage	rs1351111	*KLRC4-KLRK1*	−0.34	4.6 × 10^−15^	NA	NA	blood + skin promoter
macrophage	rs1351111	*KLRK1*	−0.35	1.5 × 10^−14^	NA	adipose, fibroblast, muscle	blood + skin promoter
macrophage	rs2422631	*SIRPB1*	−0.40	2.2 × 10^−13^	NA	WB, lung, nerve	blood DNAase
macrophage	rs28383372	*HLA-DQA2*	0.25	2.2 × 10^−13^	NA	WB, lung, adipose	5+-tissue promotor
macrophage	rs866865	*KCNMA1*	−0.30	3.7 × 10^−13^	IFN-g, rs752372	WB, adipose, lung	blood enhancer
macrophage	rs2278589	*MARCO*	−0.40	1.8 × 10^−12^	NA	adipose, WB, skin	adipocytes, monocytes
macrophage	rs1063347	*HLA-DQB1*	−0.29	7.9 × 10^−12^	NA	WB, lung, skin	blood promoter + histone marks
macrophage	rs634512	*LYZ*	−0.27	2.2 × 10^−11^	LPS24, rs1384	WB, lung, artery	blood promoter. breast/skin DNAase
macrophage	rs4528348	*MS4A14*	0.26	5.4 × 10^−11^	LPS24, rs2233253	adipose, lung, nerve	blood promoter + enhancer liver/lung enhancer + DNAase
macrophage	rs2327276	*VNN2*	−0.34	7.8 × 10^−11^	IFN-g, rs1883613	adipose, WB, lung	blood + skin promoter
endothelial	rs28383362	*HLA-DQA2*	−0.34	1.5 × 10^−22^	NA	WB, muscle, adipose	blood promoter + enhancer
endothelial	rs1884841	*TC2N*	−0.23	1.0 × 10^−12^	NA	nerve, adipose, artery	blood DNAse + enhancer
endothelial	rs182366	*B3GALNT2*	0.26	7.0 × 10^−12^	NA	nerve, adipose, thyroid	blood promoter, skin DNAase
endothelial	rs2744944	*UHRF1BP1*	−0.26	8.16 × 10^−12^	NA	artery, muscle, fibroblast	blood enhancer
endothelial	rs2977786	*DEFB1*	−0.27	4.3 × 10^−11^	NA	adipose, heart, nerve	5-+ tissue promotor
endothelial	rs61799378	*SLC25A24*	−0.28	1.5 × 10^−10^	NA	testis, WB, fibroblast	blood promoter
endothelial	rs4728142	*IRF5*	−0.28	3.2 × 10^−10^	NA	WB, artery, thyroid	blood/fat promoter
endothelial	rs9270111	*HLA-DRB5*	0.30	6.3 × 10^−10^	NA	muscle, WB, adipose	blood cell enhancer
endothelial	rs3819715	*HLA-DQB1*	−0.35	9.0 × 10^−10^	NA	adipose, muscle, skin	blood cell enhancer
endothelial	rs3760516	*VAMP2*	−0.33	9.6 × 10^−10^	NA	nerve, brain, thyroid	blood promoter + enhancer
adipocyte	rs28383362	*HLA-DQA2*	0.26	4.2 × 10^−13^	NA	WB, muscle, adipose	blood promoter
adipocyte	rs2977786	*DEFB1*	0.27	4.6 × 10^−11^	NA	adipose, heart, nerve	blood promoter
adipocyte	rs1812350	*B3GALNT2*	−0.25	6.1 × 10^−11^	NA	nerve, adipose, thyroid	blood promoter
adipocyte	rs1884841	*TC2N*	0.20	6.2 × 10^−10^	NA	nerve, adipose, artery	heart/muscle promoter

The first column, “Cell Type,” lists the cell-type proportion estimate included in the G × cell-proportion interaction model. Macrophage proportion interactions replicated in Fairfax et al., 2015^40^ have proxy SNPs and stimuli condition annotated. The top three eQTL tissues in GTEx are listed on the basis of effect size. The “Regulatory Regions” column lists HaploRegv4 annotations at the lead SNP. All promoters, enhancers, and other regulatory annotation enrichments are derived from HaploRegv4.

Five macrophage-dependent eQTLs were replicated in a context-specific monocyte eQTL dataset.^41^ Four of the five were detected in an *IFN*-γ- or LPS-challenged state. Overall, the lead G × cell-type-proportion SNPs were enriched for overlap with HaploReg enhancer annotations in primary monocytes (p value = 0.001) and neutrophils (p value = 0.004), consistent with the large number of G × cell eQTLs dependent on macrophage proportion (60%).

We intersected all 26 significant G × cell interactions with genome-wide significant (GWS) associations in the NHGRI GWAS catalog and identified nine G × cell lead SNPs that overlap with GWAS variants or are in strong linkage disequilibrium (LD) (r^2^ > 0.80, D’ > 0.9) with GWS loci. Seven out of nine of these SNPs are within the MHC and are coincident with multiple immune- and autoimmune-disorder GWAS loci. The seven G × cell interaction SNPs in the MHC appear to represent independent signals, and only two (rs28383362 and rs28383372, r^2^ = 0.66) showed a pairwise r^2^ greater than 0.51. Two MHC genes, *HLA-DRB5* [MIM: 604776] and *HLA-DBQ1* [MIM: 604305], had interactions with both the endothelial and the macrophage proportion at two different SNPs that are not in LD (*HLA-DRB5*-rs9270111 and rs28383362 r^2^ = 0.1; *HLA-DBQ1*-rs1063347 and rs3819715 r^2^ = 0.003), indicating that different SNPs regulate these genes in the different cell types. The two non-MHC GWAS coincident G × cell interaction SNPs are rs1351111, which is coincident with GWAS lead SNPs for Behcets disease (r2 = 1; rs2617170) and whose regulation of *KLRK1* [MIM: 611817] is dependent on macrophage proportion,^42^ and rs4728142, whose regulation of *IRF5* [MIM: 607218] is dependent on endothelial proportion and which is the lead SNP in GWASs for a range of auto-immune diseases including ulcerative colitis and systemic lupus erythematous.^43^, ^44^

### Cell Types Are Not Associated with BMI Genetic-Risk Scores

To understand whether adipose cell-type variability was due to the genetic control of BMI, we sought to determine whether any of our cell proportion estimates were associated with BMI genetic-risk scores (GRSs). First, we calculated weighted BMI GRSs on the full set of TwinsUK-genotyped individuals. For the GRS calculations, we used the 941 BMI SNPs reported in the latest BMI GWAS meta-analysis.^45^ Of these, 926 were present in our QC-ed imputation. Betas and effect alleles were extracted from meta-analysis summary statistics. We note that the GRS are positively associated with median BMI (median of all longitudinal measurements) in the full TwinsUK sample (beta = 3.6; p value = < 2.2 × 10^−16^. R^2^ for median BMI residuals adjusted for all other covariates ≈1.7%, n = 6K). To assess whether BMI GRS is associated with macrophage infiltration or in fact any cell type estimated in these analyses, we fit linear mixed models accounting for twin relatedness and age. We find no association between BMI GRS and cell estimates (all p value > 0.05); this suggests that cell proportion variation isn’t driven by genetically influenced obesity, and it is therefore likely to be a secondary effect of becoming overweight or obese.

## Discussion

RNA-seq profiling of bulk-tissue biopsies is widely used for biomarker discovery, genetics of gene expression studies, and differential expression analysis^5^, ^9^, ^20^, ^40^ but the cellular complexity of primary-tissue biopsies is often unaccounted for. In this study, we used *in silico* methods to characterize the variability of adipose cell-type composition in two large bulk-tissue transcriptomic datasets and explored the effects of adipose cellular heterogeneity on a range of transcriptomic analyses. Our results indicate that it is critical to account for cell-type composition when combining adipose transcriptome datasets in co-expression analysis and in differential expression analysis with obesity-related traits.

Although the ability to detect interactions with estimated cell proportions is limited in terms of both sample size and the accuracy of cell-type estimation from a complex tissue such as adipose, we have demonstrated that its possible to detect cell-type-proportion-dependent eQTLs in whole adipose tissues. We identified 26 macrophage-, endothelial-, or adipocyte-specific eQTLs within our bulk adipose tissue RNA-seq datasets, and we note that all of these had main-effect eQTLs in TwinsUK adipose tissue and in several GTEx tissues (Table 2). The presence of immune- and endothelial-specific eQTLs is expected in other tissues with resident immune cells and blood vessels, however, three of the four adipocyte-dependent eQTLs have been found to be eQTLs in GTEx nerve tissue. Adipose tissue is spread throughout the body and around organs, and obtaining adipose-free biopsies of many tissues, including nerve, thyroid, and muscle tissues, is technically difficult, as is clearly documented in the GTEx pathologist notes and histology slides that are provided for every biopsy. Our conjecture is that the presence of adipocyte-specific eQTLs in nerve tissue is a result of adipose contamination of the nerve biopsies. This suggests that estimates of tissue sharing of expression or regulatory effects between adipose and some tissues are likely to be an overestimated.^46^ It is thus important to consider the cell-type composition of biopsies prior to utilizing expression or eQTL data to interpret disease *loci*, and in particular before prioritizing a tissue or cell type for downstream experiments.

Several shortcomings of our study are worth mentioning for future improvements. We have estimated the relative proportion of cell types in two biopsy datasets, and it is important to note that the content of a biopsy might not be representative of the cell content of the *in vivo* tissue from which it was extracted. Many technical factors, including method of retrieval (surgical biopsy versus lipoaspiration versus needle biopsy) and sample handling (as demonstrated by the association between cell-type heterogeneity and ischemic time in GTEx), are known to influence adipose biopsy composition. Second, CIBERSORT estimates relative fractions of cell types, not absolute proportions. This means the cell-type proportion estimates are only interpretable relative to what is included in the signature matrix and should not be interpreted as absolute proportions of those cell types *in vivo*. Finally, a broader and better-defined signature matrix would increase both accuracy and utility of the method. We did not include additional adipose-resident cell types such as adipose mesenchymal stem cells, pre-adipocytes, and a wider range of lymphocytes due to the lack of suitable available reference RNA-seq datasets. In particular, the lack of reference adipose mesenchymal stem cells and pre-adipocytes could inflate our estimates of the proportion of adipocytes (the cell type most closely correlated with these cell types) relative to the other cell types in the matrix. We expect the utility of deconvolution of bulk-tissue gene expression to further improve as more RNA-seq datasets of primary and purified cells become available.

We have shown *in silico* deconvolution to have strong utility for understanding how cell-type proportions vary in population studies of adipose tissue. We demonstrate that adipose-cell composition is heritable and associated with body-fat distribution. Although some of this heritability might be mediated by overall BMI heritability, which in turn might drive changes in cell composition, it is possible that certain genotypes could predispose individuals to or protect them from macrophage infiltration and thereby the consequences of inflammation in obesity. Heritable variability in adipocyte number could also underlie differential capacity for adipose-tissue expansion and storage, which can drive ectopic fat deposition and subsequent susceptibility to downstream cardio-metabolic disease. The role of cellular heterogeneity in modulating human health and disease is a growing area of interest,^12^ and further deconvolution of bulk RNA-seq datasets, aided by the expanding availability of RNA from primary and iPSC-derived cell populations and single-cell analysis, should contribute to our understanding of how genetics influence cell-type heterogeneity and its impact on health and disease.

## Declaration of Interests

The authors declare no competing interests.

## Web Resources

Data and summary statistics can be found here, https://github.com/GlastonburyC/CellTypeDeconvolution
CIBERSORT, https://cibersort.stanford.edu/
Database of Genotypes and Phenotypes (dbGAP), https://www.ncbi.nlm.nih.gov/gap
Genotype-Tissue Expression project (GTEx) Portal, https://gtexportal.org/home/
Online Mendelian Inheritance in Man, https://www.omim.org/

## Supplemental Data

Document S2. Tables S1, S2, and S4

Document S2. Article plus Supplemental Data
