# Supplementary material for: Cell-Type Heterogeneity in Adipose Tissue Is Associated with Complex Traits and Reveals Disease-Relevant Cell-Specific eQTLs
Source: Am J Hum Genet. 2019 May 23;104(6):1013–24. doi: 10.1016/j.ajhg.2019.03.025 (PMC6556877; doi:10.1016/j.ajhg.2019.03.025)
Supplement: Document S1. Figures S1–S7, Tables S3, S5, and S6 [file mmc1.pdf]

The American Journal of Human Genetics, Volume *104*

Supplemental Data

Cell-Type Heterogeneity in Adipose Tissue Is Associated with Complex Traits and Reveals Disease-Relevant Cell-Specific eQTLs

Craig A. Glastonbury, Alexessander Couto Alves, Julia S. El-Sayed Moustafa, and Kerrin S. Small

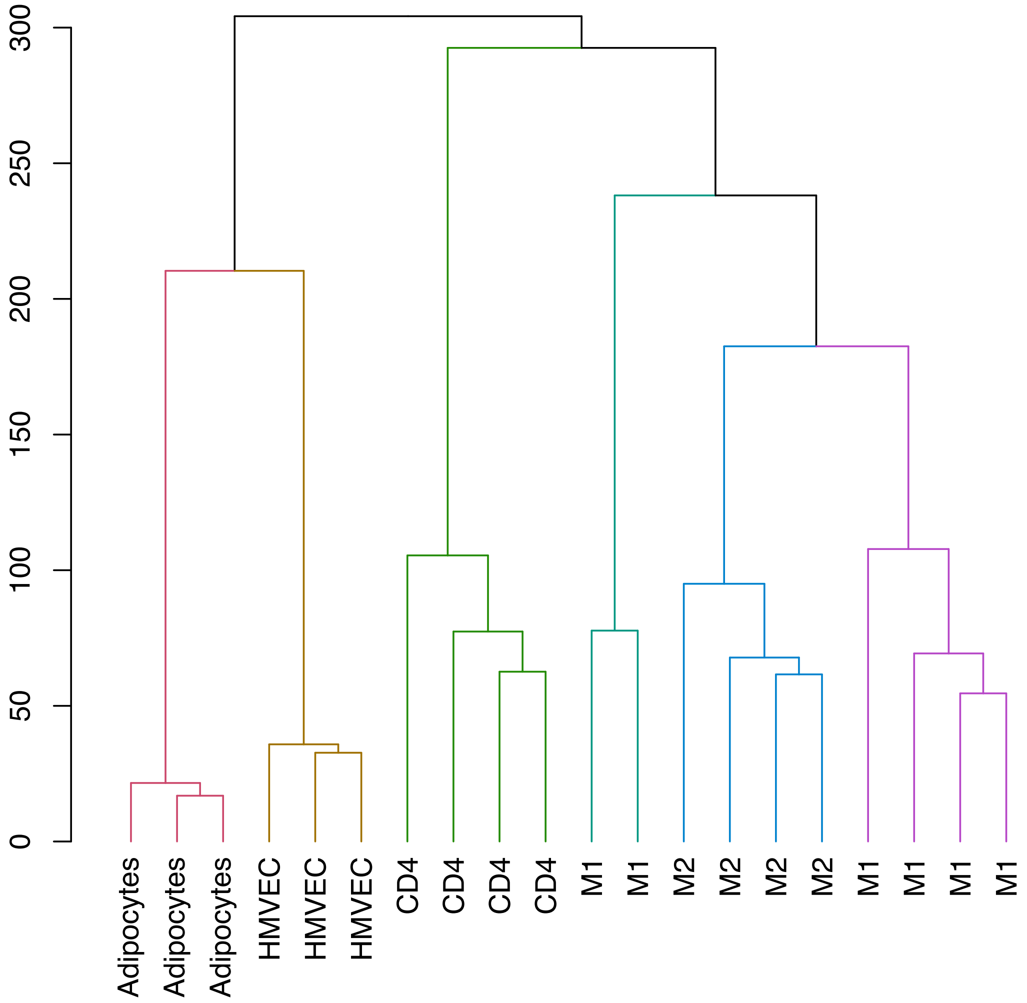

Figure S1: **Hierarchical clustering of reference cells that are used to produce the signature matrix.** Coloured by unsupervised k-means clustering (where k = 5). Biological hierarchy recapitulated: Non-immune (Adipocytes, MVEC) and immune cell fractions (Macrophage and CD4+ t-cells) cluster separately

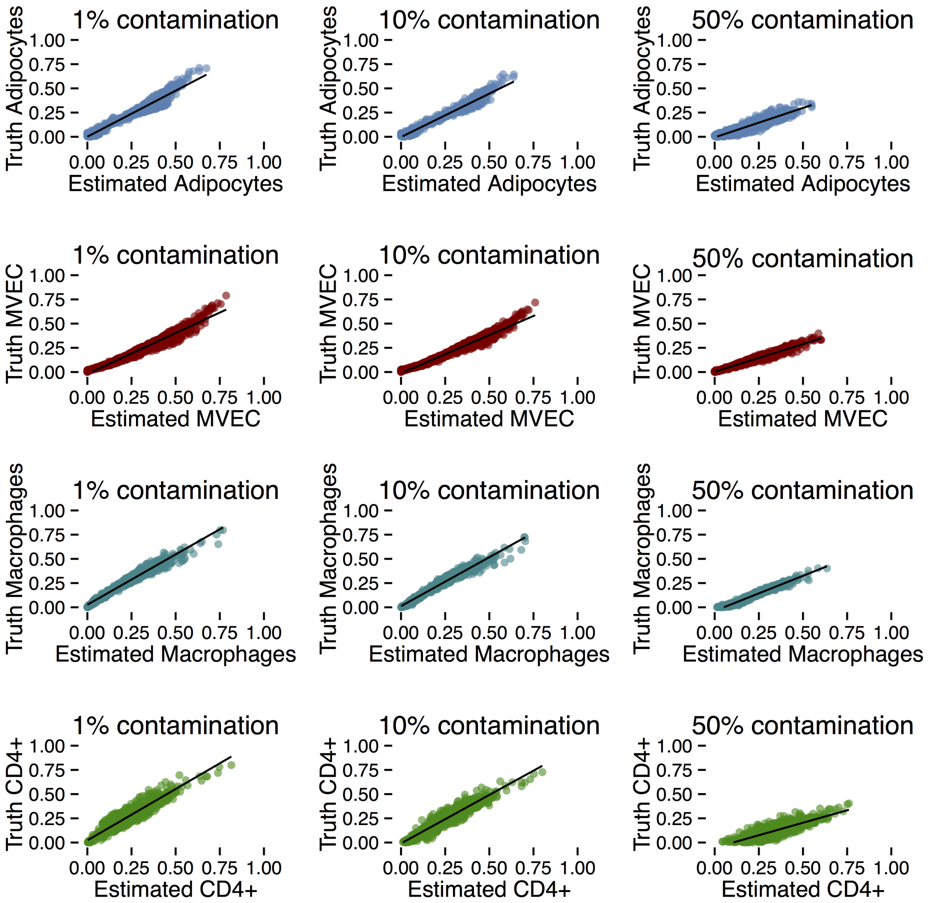

Figure S2: **Effect of *In-silico* cell type estimation with unknown content added.** Additional cell types known to be present in adipose tissue (Fibroblasts, Neutrophils & Dendritic Cells) but that are not estimated by the adipose signature matrix, were included in simulated adipose tissue mixtures (Adipocytes, CD4+, MVEC, Macrophages) to assess estimation accuracy with varying amounts of unknown content. The adipose tissue signature matrix is robust to unknown cell types, with cell estimates maintaining a highly linear relationship with ground truth data. An unlikely scenario of 50% unaccounted for mixture content, resulted in systematic overestimation, yet a linear relationship was still maintained.

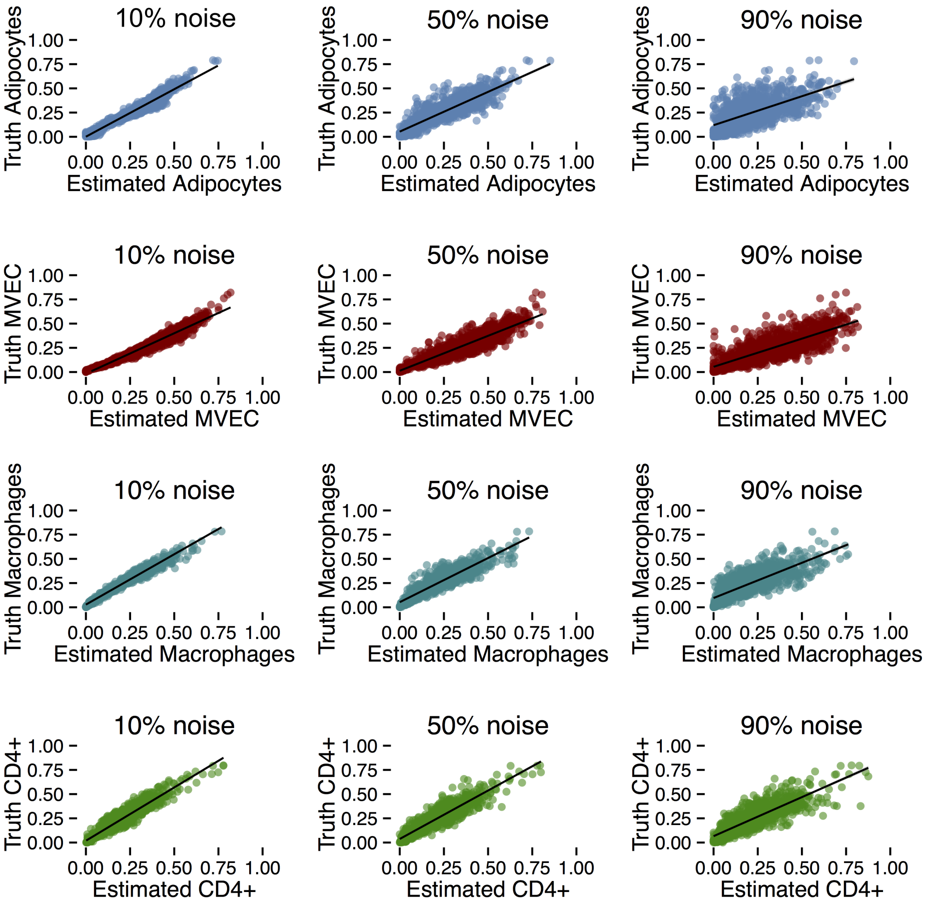

Figure S3: **Effect of noise on deconvolving cell types.** Cells type estimates from *in-silico* simulations with added scaled Gaussian noise (10, 50, 90% respectively).

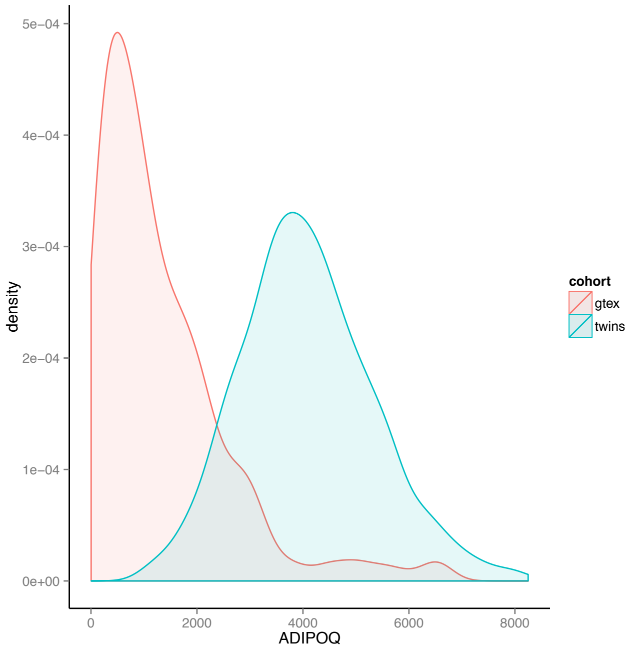

Figure S4: **Distribution of ADIPOQ expression.** Expression of ADIPOQ (TMM) in TwinsUK and GTEx samples have strikingly different distributions.

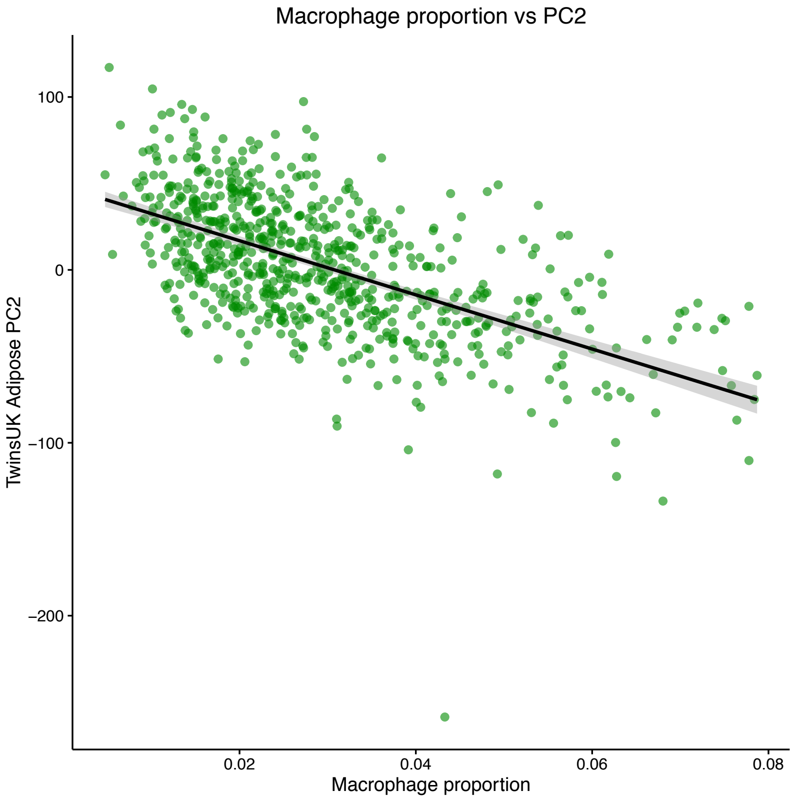

Figure S5: **Macrophage proportion vs gene expression PCs.** Adipose tissue RNA-seq PC2 captures macrophage proportion heterogenity amongst samples.

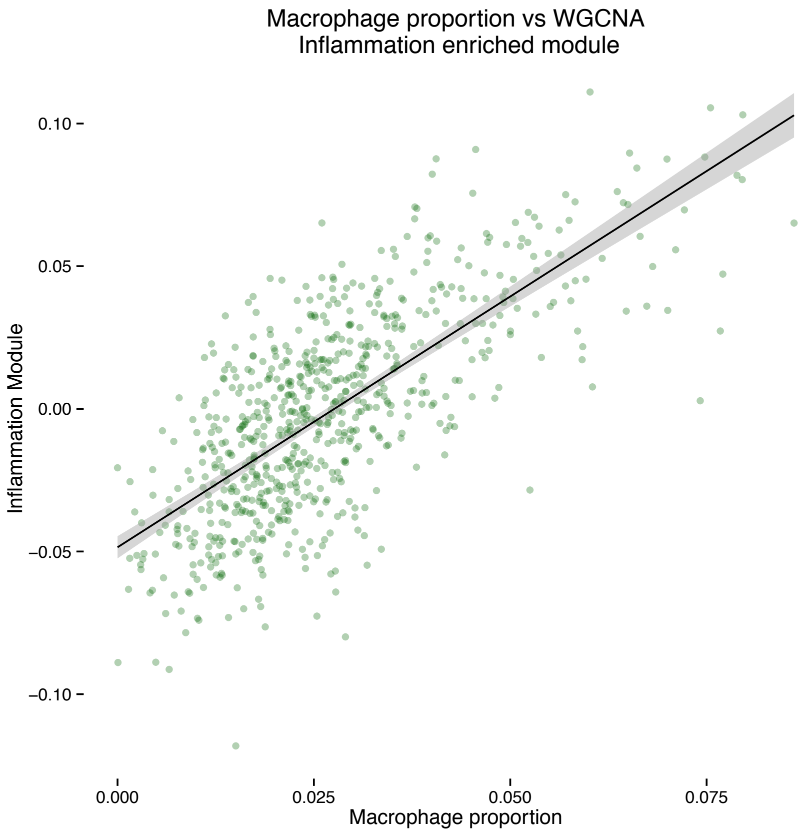

(A) Correlation of the inflammation enriched module with estimated macrophage proportion.

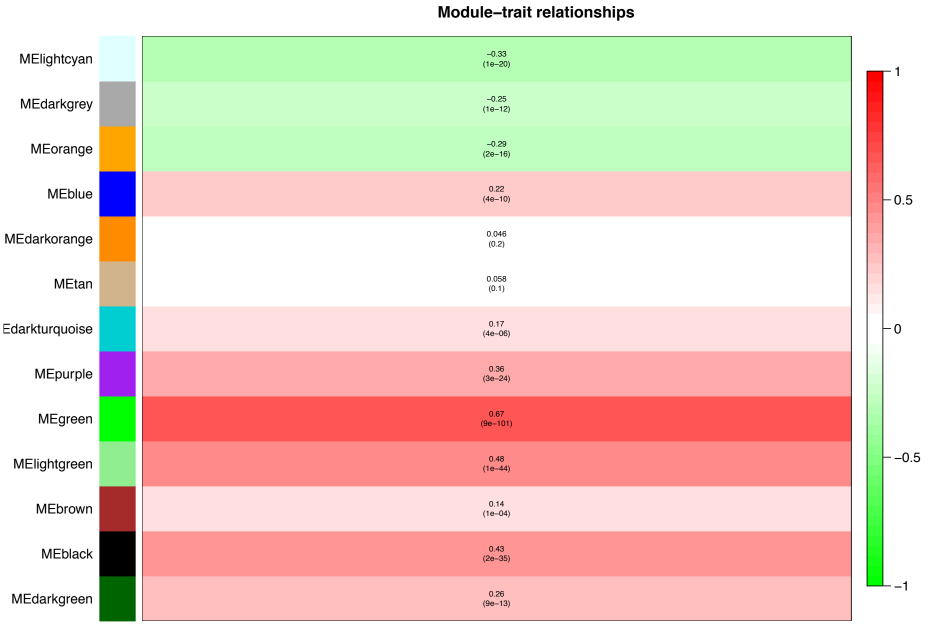

(B) Correlation of macrophage proportion with all WGCNA modules.

Figure S6: **WGCNA modules and their correlation to macrophage proportion in subcutaneous adipose tissue.**

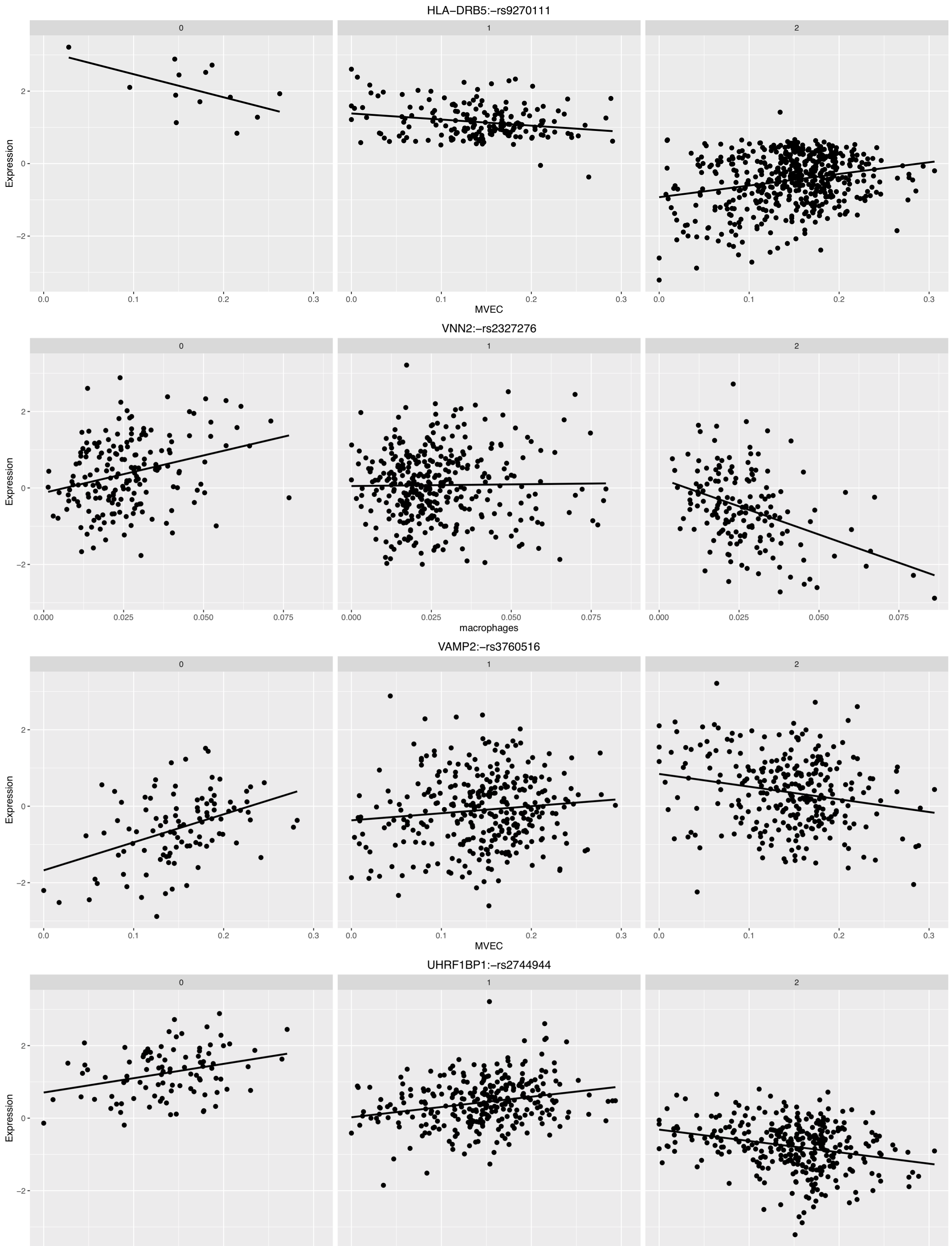
HLA−DRB5:−rs9270111
0
1
2
Expression
MVEC
VNN2:−rs2327276
macrophages
VAMP2:−rs3760516
UHRF1BP1:−rs2744944

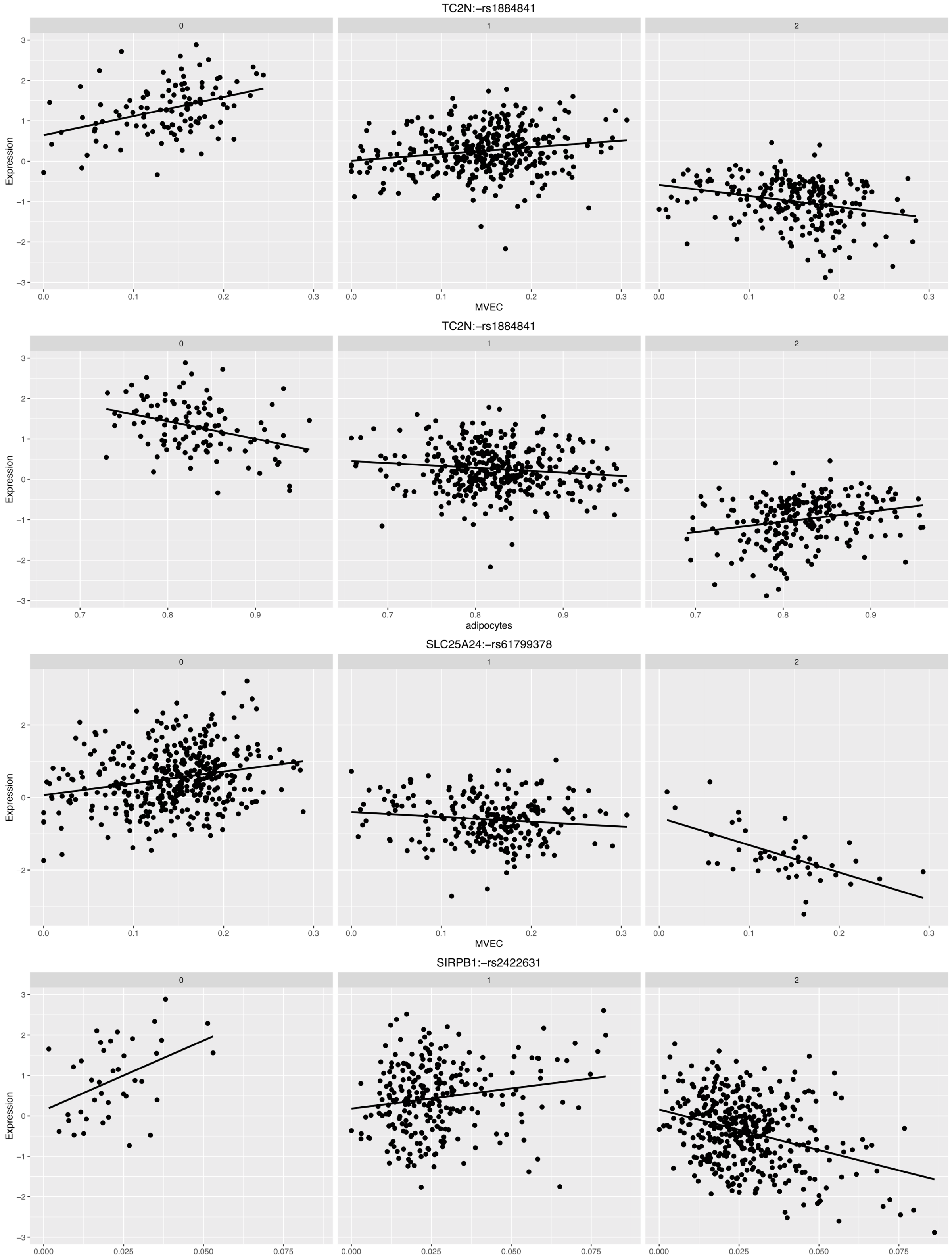

TC2N:−rs1884841
0
1
2
Expression
MVEC
TC2N:−rs1884841
0
1
2
Expression
adipocytes
SLC25A24:−rs61799378
0
1
2
Expression
MVEC
SIRPB1:−rs2422631
0
1
2
Expression
macrophages

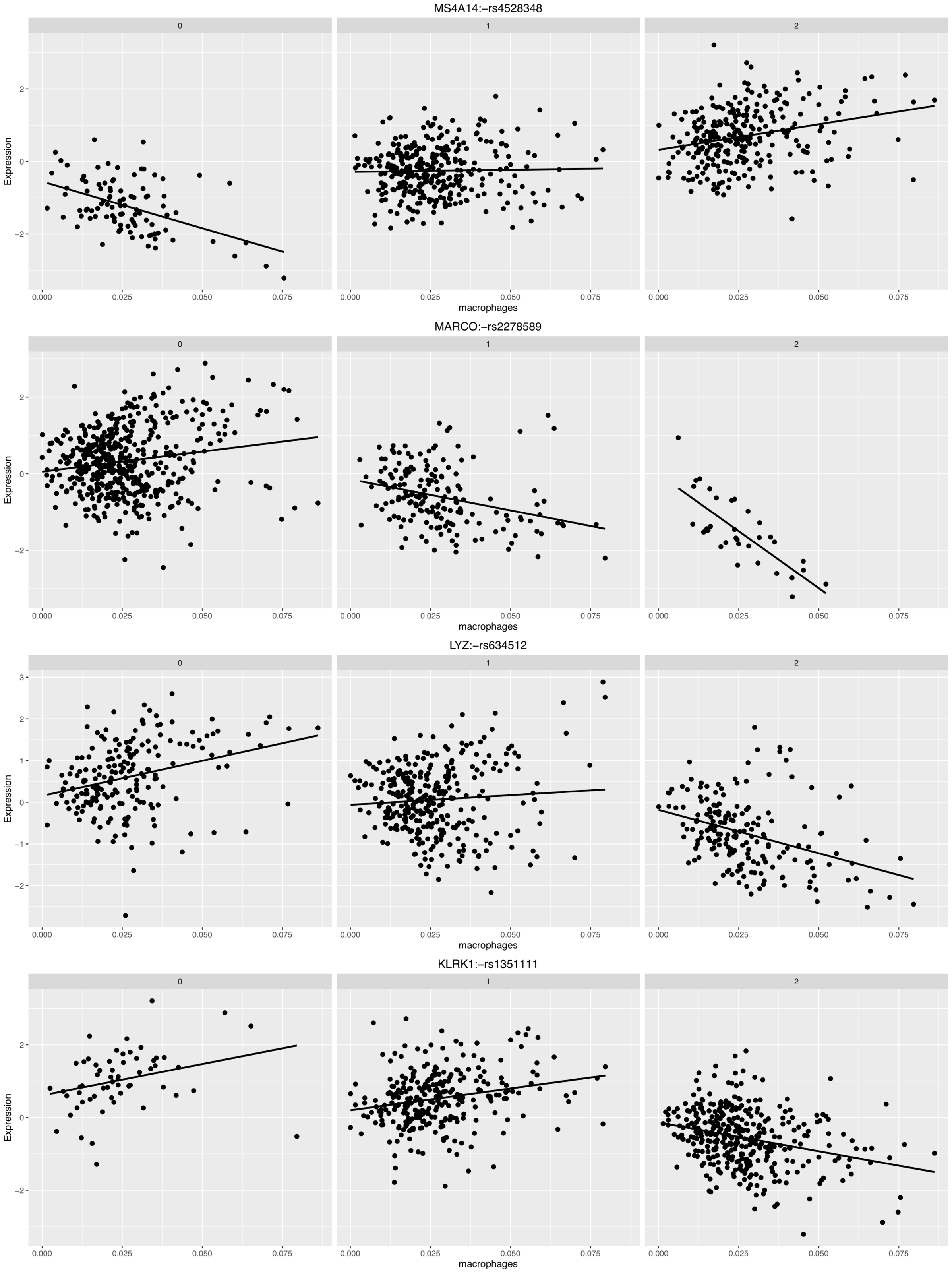
MS4A14:-rs4528348
MARCO:-rs2278589
LYZ:-rs634512
KLRK1:-rs1351111
Expression
macrophages
0
1
2

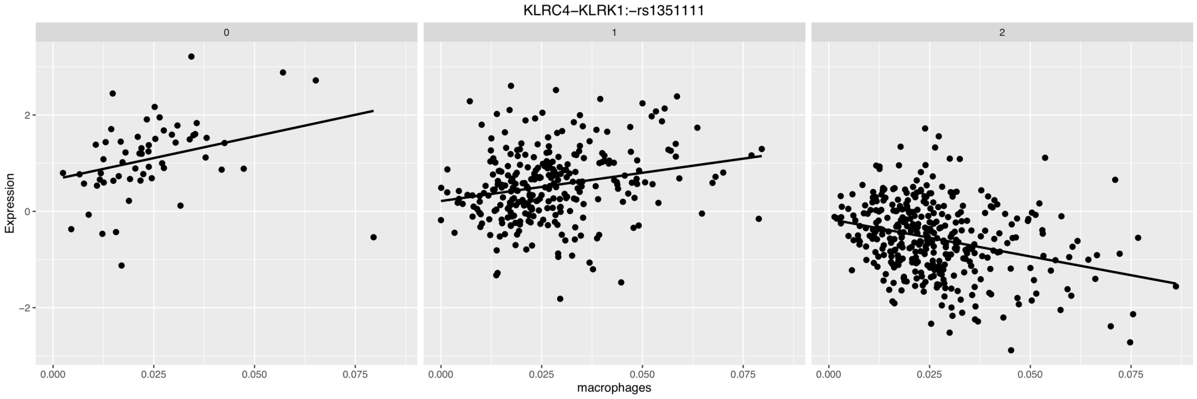
KLRC4−KLRK1:−rs1351111
0
1
2
Expression
macrophages
2
0
−2
0.000
0.025
0.050
0.075

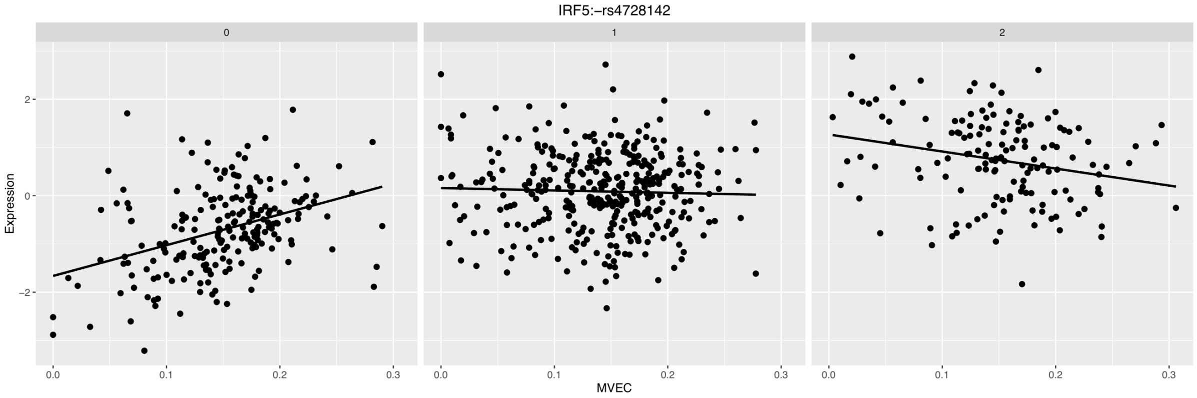
IRF5:−rs4728142
0
1
2
Expression
MVEC
2
0
−2
0.0
0.1
0.2
0.3

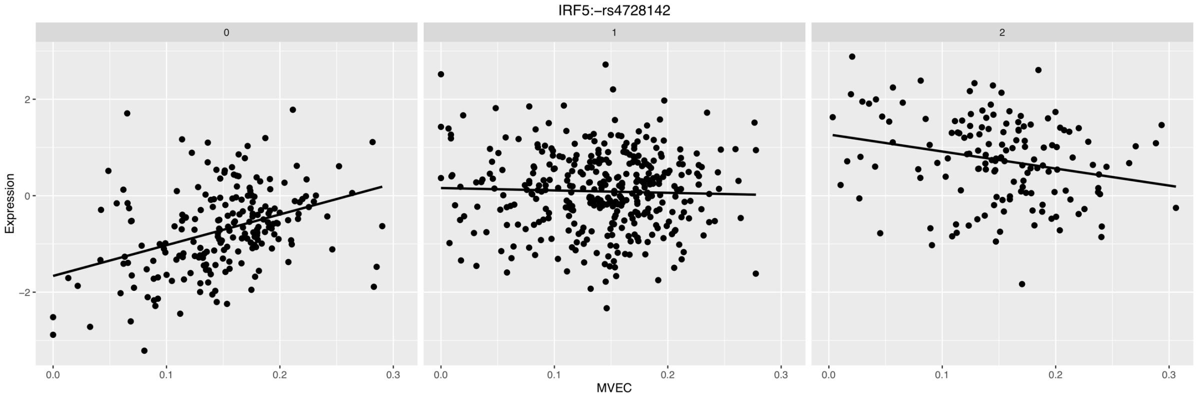
IRF5:−rs4728142
0
1
2
Expression
MVEC
2
0
−2
0.0
0.1
0.2
0.3

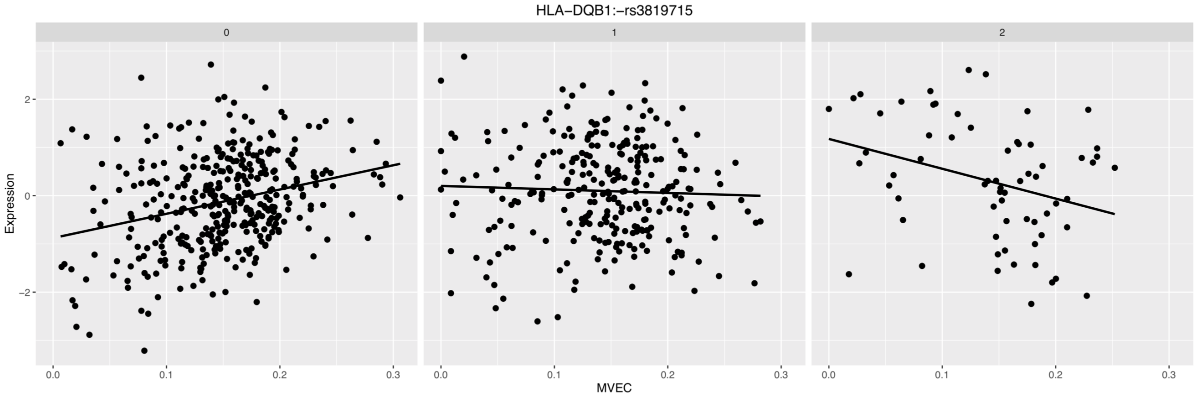
HLA−DQB1:−rs3819715
0
1
2
Expression
MVEC
2
0
−2
0.0
0.1
0.2
0.3

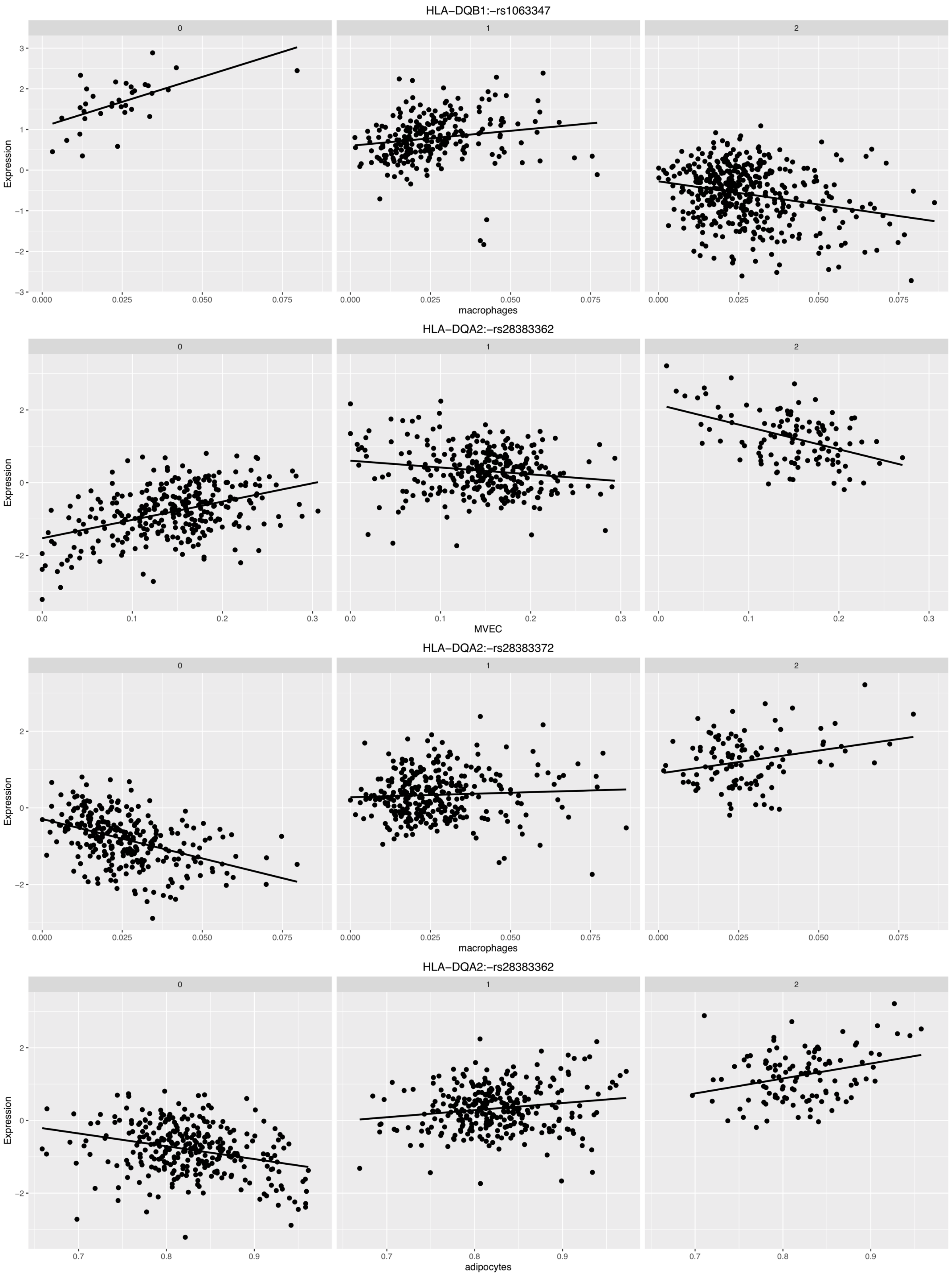
HLA−DQB1:−rs1063347
0
1
2
Expression
macrophages
HLA−DQA2:−rs28383362
0
1
2
Expression
MVEC
HLA−DQA2:−rs28383372
0
1
2
Expression
macrophages
HLA−DQA2:−rs28383362
0
1
2
Expression
adipocytes

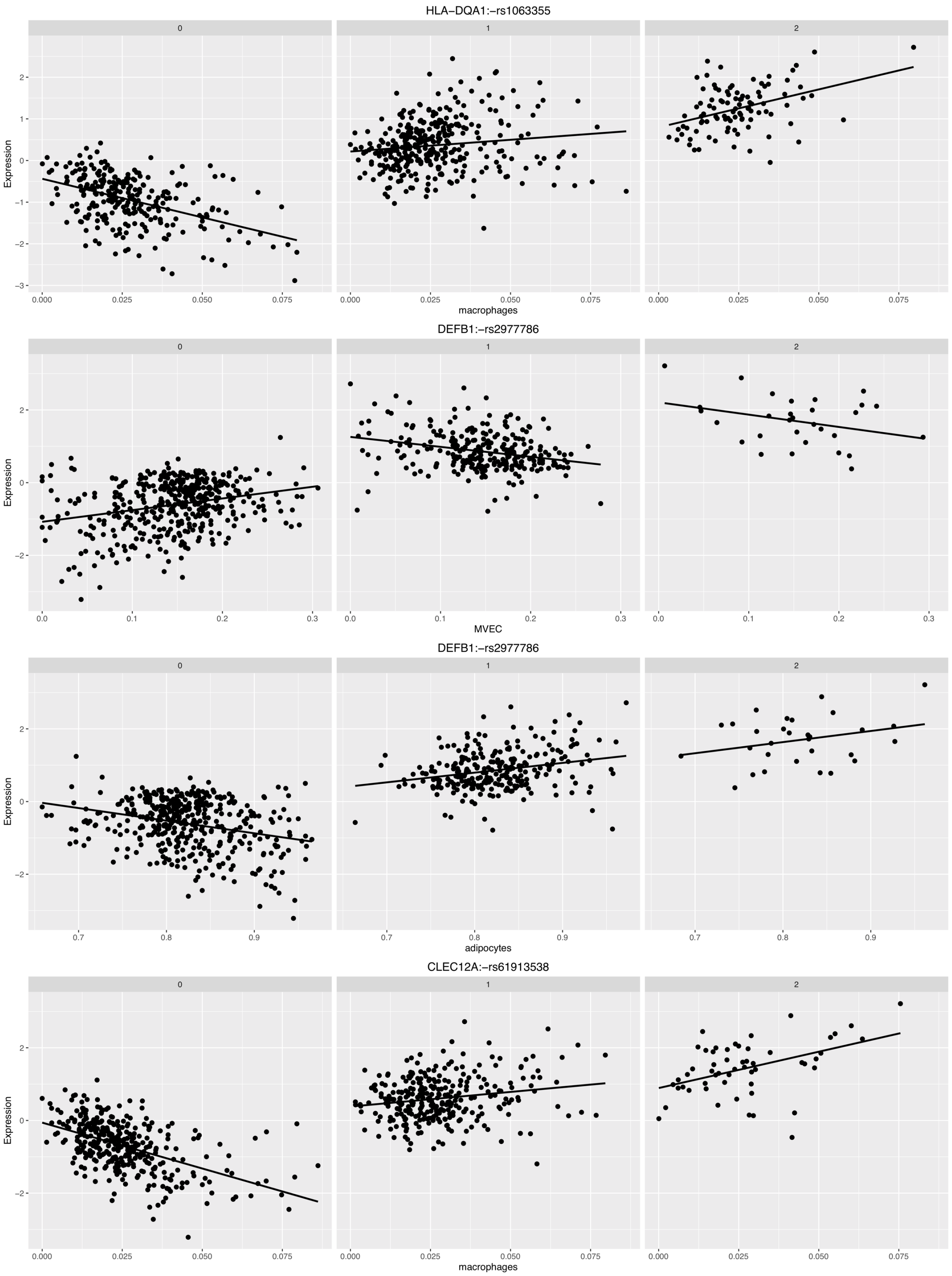
HLA−DQA1:−rs1063355
0
1
2
Expression
macrophages
DEFB1:−rs2977786
0
1
2
Expression
MVEC
DEFB1:−rs2977786
0
1
2
Expression
adipocytes
CLEC12A:−rs61913538
0
1
2
Expression
macrophages

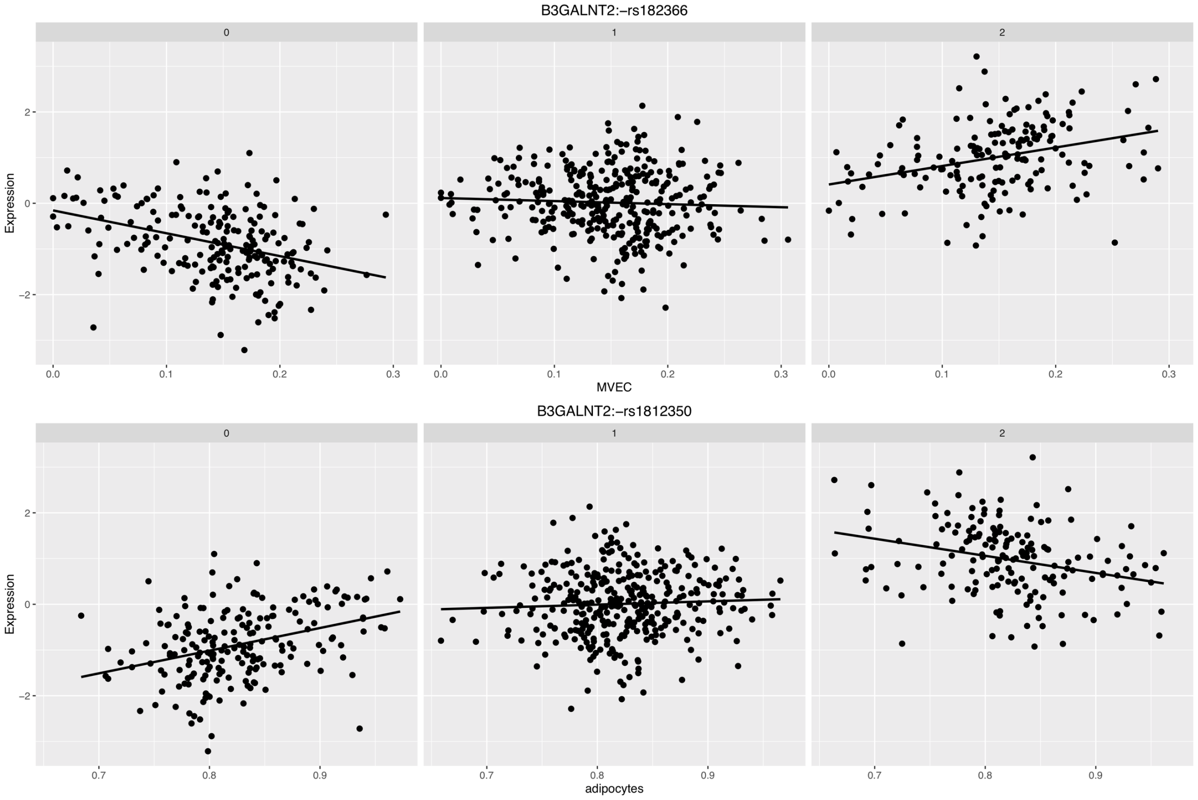

Figure S7: **All 26 GxCell interactions plotted.** Cell proportion (x-axis) and gene expression (y-axis) seperated by genotype dosage (0, 1, 2)

Cell type	Citation	SRA accession
White adipocyte	(Moisan et al. 2015)	SRR1296133, SRR1296134, SRR1296135
T-cell (CD4+)	(Weinstein et al. 2014)	SRR1422906, SRR1422907, SRR1422908, SRR1422909
HMVEC	(DiMaio et al. 2016)	SRR2776477, SRR2776478, SRR2776479
M1/M2 macrophages	(Zhang et al. 2015)	SRR2910670, SRR2910671, SRR2939145, SRR2939146, SRR2939148, SRR2939149, SRR2939150, SRR2939151, SRR2939152

Table S3: **Dataset sources for signature matrix.** Purifed cell RNA-seq data from SRA used to construct Adipose tissue signature matrix

Cell type	Adipocytes (%)	CD4+ t-cell (%)	MVEC (%)	M1 Macrophage (%)	M2 Macrophage (%)
**Adipocytes**	**99.5**	0.003	0	0.001	0
**CD4+ t-cell**	0	**100**	0	0	0
**MVEC**	0.9	0	**98.8**	0	0
**Macrophage**	0	**5.6**	0	**1**	**93.3**

Table S5: **Estimates of purified cell types**. Cell type (%) estimates when applying the adipose tissue signature matrix to four independent samples of purified cells. Top row represents cells present in adipose tissue signature matrix. Left most column represents independent set of purified cell RNA-seq profiles.

Study	Cell type	Proportion (%)	Sample size	Sex	Age	Correlated with adiposity?
	CD4+	3-4.7	17	M	35-55	n.s
Travers et al. (2015)	CD8+	0.5-5.7	-	-	-	n.s
	Macrophages	2.9 - 15.5	-	-	-	P <0.05 (+)
Zimmerlin et al. (2010)	Endothelial cells	15.4 ± 4.8	8	F	-	not tested
Van Harmelen et al. (2003)	Adipocytes	85	49	M/F	16-73	P <0.05 (+)

Table S6: **Previous published estimates of adipose cell types.** Estimates from several studies that flow sorted subcutaneous adipose tissue to measure cell proportions and their relationship with adiposity.
